# Shifting the paradigm in personalized cancer care through next‐generation therapeutics and computational pathology

**DOI:** 10.1002/1878-0261.13724

**Published:** 2024-08-30

**Authors:** Jorge S. Reis‐Filho, Maurizio Scaltriti, Ansh Kapil, Hadassah Sade, Susan Galbraith

**Affiliations:** ^1^ Cancer Biomarker Development, Oncology Research and Development AstraZeneca Gaithersburg MD USA; ^2^ Translational Medicine, Oncology Research and Development AstraZeneca Gaithersburg MD USA; ^3^ Oncology Research and Development AstraZeneca Computational Pathology GmbH, AstraZeneca Munich Germany; ^4^ Oncology Research and Development AstraZeneca Gaithersburg Maryland USA

**Keywords:** antibody drug conjugates, artificial intelligence, biomarkers, computational pathology, deep learning, digital pathology

## Abstract

The incorporation of novel therapeutic agents such as antibody‐drug conjugates, radio‐conjugates, T‐cell engagers, and chimeric antigen receptor cell therapies represents a paradigm shift in oncology. Cell‐surface target quantification, quantitative assessment of receptor internalization, and changes in the tumor microenvironment (TME) are essential variables in the development of biomarkers for patient selection and therapeutic response. Assessing these parameters requires capabilities that transcend those of traditional biomarker approaches based on immunohistochemistry, *in situ* hybridization and/or sequencing assays. Computational pathology is emerging as a transformative solution in this new therapeutic landscape, enabling detailed assessment of not only target presence, expression levels, and intra‐tumor distribution but also of additional phenotypic features of tumor cells and their surrounding TME. Here, we delineate the pivotal role of computational pathology in enhancing the efficacy and specificity of these advanced therapeutics, underscoring the integration of novel artificial intelligence models that promise to revolutionize biomarker discovery and drug development.

## Graphical abstract

The evolution from traditional pathology methods to computational pathology technologies enables the development of transformational biomarkers for patient selection and therapeutic response prediction, maximizing the potential of personalized cancer care.

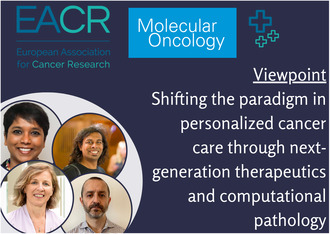

**Abbreviations**
ADCs: antibody‐drug conjugates
AI: artificial intelligence
CAR‐T: chimeric antigen receptor T‐cell
DL: deep learning
H&E: hematoxylin & eosin
HER2: human epidermal growth factor receptor 2
HRD: homologous recombination‐deficiency
IHC: immunohistochemistry
ML: machine learning
MSI: microsatellite instability
NSCLCs: non‐small cell lung cancers
PD‐L1: programmed death‐ligand 1
QCS: quantitative continuous score
RICs: radioimmunoconjugates
TCEs: T‐cell engagers
TILs: tumor‐infiltrating lymphocytes
TLSs: tertiary lymphoid structures
TME: tumor microenvironment
WSIs: whole slide images

## 1 Introduction

The unparalleled advancements of drug development provided by the advent of sophisticated biological agents, including antibody‐drug conjugates (ADCs), radioimmunoconjugates (RICs), T‐cell engagers (TCEs), and chimeric antigen receptor (CAR‐T) therapies have brought precision oncology at an inflection point. To guide the delivery of these transformational agents, traditional approaches (e.g., massively parallel sequencing or conventional pathology methods) no longer suffice. Rather, these novel therapeutic modalities have posed fundamental challenges in biomarker development.

Traditional diagnostic modalities that have long been one of the cornerstones for biomarker assessment, such as conventional immunohistochemistry (IHC), have inherent limitations related to their sensitivity, specificity and dynamic range, as well as the well‐known intra‐ and inter‐observer variability stemming from visual assessment, even when performed by trained pathologists. For these novel therapeutic agents, the detection of the respective targets in the correct subcellular compartment (e.g., cell membrane), as well as the precise assessment of their abundance and distribution are essential. Furthermore, information contained in the tumor microenvironment (TME) composition and distribution may offer additional biological insights and potentially predictive information that can help define the patients who will benefit the most from these new and transformative treatments.

To deliver on the promise of this new wave of cancer therapeutics, a paradigm shift toward new methodologies that elucidate more than just the presence of a target is needed. Accurately quantifying and spatially resolving the expression of therapeutic targets within specific cellular and subcellular compartments are required to gain additional biological insights from cancer cells and their TME.

## 2 Digital/computational pathology

**Fig. 1:**
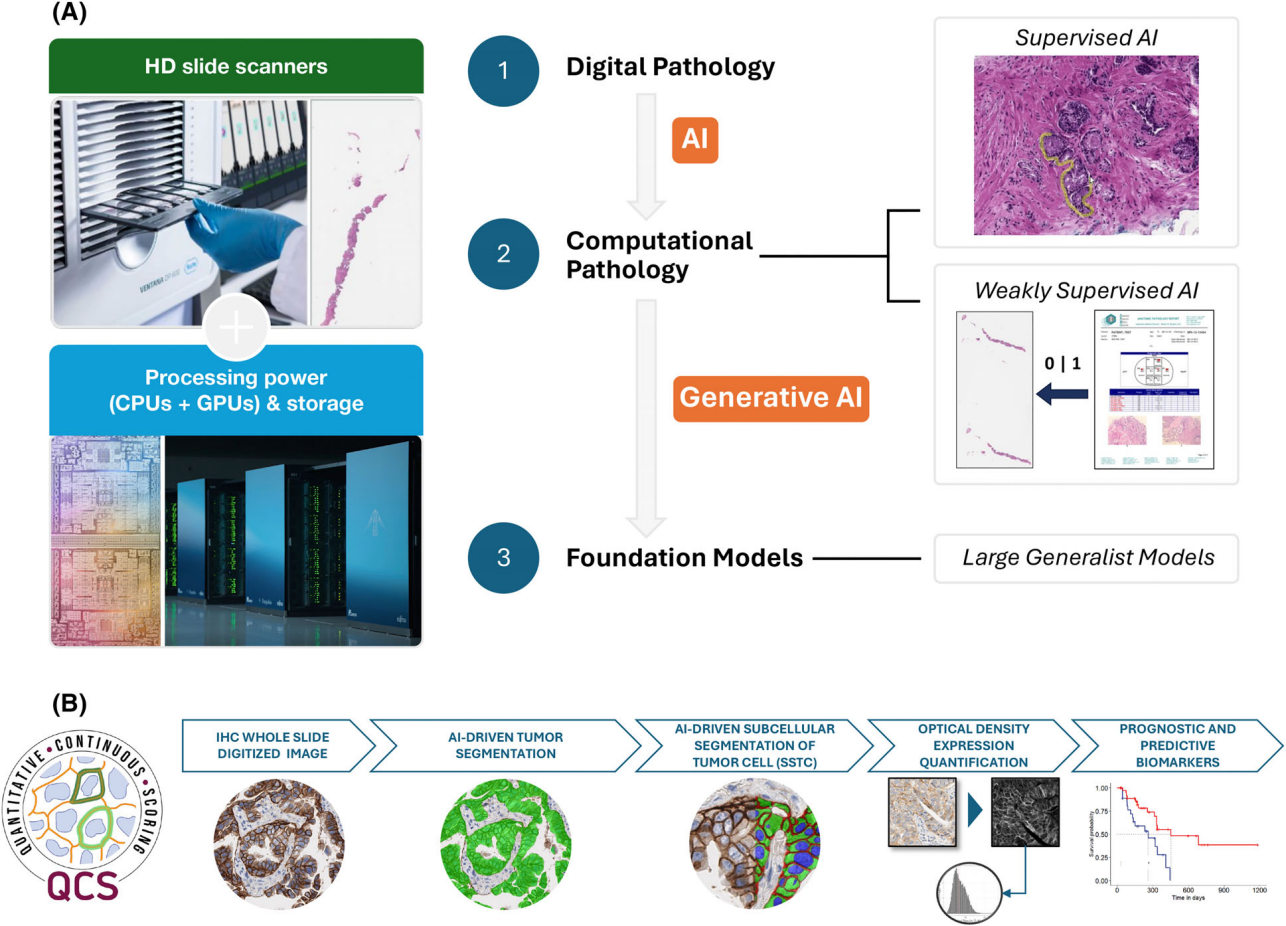
Digital and computational pathology. (A) Digital pathology has been enabled by the development of pathology slide scanners that now produce whole slide images (WSIs) of histological and immunohistochemical preparations at sufficient resolution and within acceptable turnaround times. With the availability of sufficient computer processing power and data storage, large collections of WSIs have been generated. Computational pathology solutions can be derived from the analysis of these WSIs utilizing strongly supervised artificial intelligence (AI), where the AI algorithms are trained based on annotations made by expert pathologists, or weakly supervised approaches, where deep‐learning (DL) algorithms are developed based on the labels stemming from pathology reports or clinico‐pathological characteristics ascribed to the WSIs. Strongly supervised AI‐based solutions require a more limited number of WSIs for training, less computer power and data storage, and are based on tried and tested machine learning (ML) approaches; however, these methods require labor‐intensive and time‐consuming pathology annotations performed by qualified pathologists. Conversely, weakly supervised methods bypass the need for pathology annotations, but do require substantially larger numbers of WSIs, greater computer power and data storage capabilities, and rather elaborate AI solutions. With the development of foundation models, novel opportunities for the development of biomarkers in more efficient and less data intense ways are emerging. (B) The Quantitative Continuous Score (QCS) is a strongly supervised AI solution for the precise, robust, and reproducible quantification of the expression of targets within specific subcellular compartments of cells. In addition to the optical density (OD) quantification of the target, QCS also offers objective quantification of the heterogeneity of target expression. Through integrative analyses of the expression patterns of the target in specific subcellular compartments of cancer cells, QCS can provide additional biological insights that can be utilized for the development of prognostic and predictive biomarkers.

In this context, computational pathology (Fig. 1) has emerged as a logical and likely necessary solution for the current needs in patient selection and therapeutic response prediction. Technological advancements in the performance characteristics of histology scanners computer processing power and data storage have allowed for the introduction of digital pathology in research and, more recently, in clinical practice [1, 2]. The availability of large collections of hematoxylin and eosin (H&E) and IHC whole slide images (WSIs) has provided a unique opportunity for the utilization of innovative computer vision and deep learning (DL) methods to develop computational pathology diagnostic solutions and novel biomarkers based on strongly or weakly supervised artificial intelligence (AI) approaches [1, 2] (Fig. 1).

There is burgeoning evidence to demonstrate that computational pathology solutions can offer unique opportunities to improve diagnostic accuracy, increase the efficiency of diagnostic processes, and enhance IHC‐based biomarker assessment to unprecedented levels of precision [2]. These approaches can offer automated tumor detection and segmentation, the quantification of specific biomarkers within sub‐cellular compartments with a dynamic range not attainable by visual scoring, as well as novel biological insights that can potentially be utilized for patient selection and therapeutic response prediction. For instance, computational pathology diagnostic solutions for the detection and grading of prostate and breast cancer have emerged [3, 4] and received approval by regulatory agencies [2]. Algorithms to infer biomarkers directly from H&E slides, such as microsatellite instability (MSI) status of colorectal cancers or homologous recombination‐deficiency (HRD) status of breast and ovarian cancers, have been reported [2]. More recently, computational pathology solutions based on H&E or by combining multimodal data have been developed to predict end‐to‐end outcomes of cancer patients, offering fundamentally new opportunities for biomarker development [2].

## 3 The role of computational pathology in assessing therapeutic targets

The contribution of computational pathology to tasks currently performed by pathologists transcends those stemming from DL analysis of H&Es and applied principally to the diagnostic realm. In fact, the precision, accuracy, quantitation, and dynamic range for the quantification of specific antigens detected by IHC analysis offered by computational pathology approaches have provided exciting new opportunities in the context of tissue‐based biomarker assessment.

Although the quantification of the expression of specific antigens by IHC analysis was one of the first applications of machine‐learning (ML)/DL approaches in pathology, most of the algorithms developed to date have focused on the detection and quantification of immune cell types. Quantification of tumor‐infiltrating lymphocytes (TILs), macrophages, and tertiary lymphoid structures (TLSs), together with the precise assessment of essential immune markers (e.g., PD‐L1, CD8, or CD4 cells) and their distribution in relation to the neoplastic cell compartments, can be attained with remarkably high levels of accuracy and reproducibility.

More recently, however, more accurate methods for the detection and segmentation of subcellular compartments of cancer cells with the subsequent quantification of expression of specific markers within each of the subcellular compartments have been developed. The Quantitative Continuous Score (QCS) is a strongly supervised AI solution (i.e., AI algorithms that are trained on digital images containing annotations made by expert pathologists; Fig. 1) for the detection and segmentation of the neoplastic components of tissue samples and the subsequent quantification of therapeutic targets expressed on the membrane and cytoplasm of cancer cells. QCS also offers unique opportunities to detect and quantify the presence of heterogeneity in the distribution of the target within the neoplastic compartment, and how this heterogeneity manifests itself within the tumor bulk. A hypothesis‐generating exploratory analysis of samples from patients with HER2‐low breast cancer treated with Trastuzumab‐Deruxtecan in the J101 trial provided evidence to support the notion that the dynamic range and precise quantification offered by QCS may allow for additional tailoring of the treatment in patients with HER2‐low breast cancer [5].

Given that targets can be precisely quantified in each subcellular compartment independently, QCS outputs from each of these compartments can be combined to derive additional biological insights. Another hypothesis‐generating analysis of samples from patients with non‐small cell lung cancers (NSCLCs) enrolled in the J101 trial and treated with Datopotamab‐Deruxtecan, an ADC targeting TROP2. Although the quantification of TROP2 on the cell membrane could, to a certain extent, help identify a subset of NCLC patients with a better outcome, in this study [6], the quantification of the relative levels of TROP2 on the membrane in relation to the cytoplasmic TROP2 expression (i.e., normalized membrane optical density) provided a more precise identification of the subset of NSCLCs who benefit the most from Datopotamab‐Deruxtecan [6]. Although further validation of these findings will be essential, these observations highlight how computational pathology approaches can transform IHC‐based biomarker assessment [6].

## 4 Evaluating the immune microenvironment

The characterization of the immune TME of cancers was an early application of ML/DL methods to pathology specimens; however, computational pathology is now enabling the accurate quantification of TILs and PD‐L1 in tumor and immune cells that goes beyond that attainable by visual quantification. Recent studies have shown that the quantification of PD‐L1 in cancer cells by QCS in patients with NSCLC not only surpasses the precision attained by trained diagnostic pathologists but also identifies a broader population of patients who may derive significant benefit from immunotherapy [7, 8].

Given that immune cells may play a mechanistic role in therapeutic responses to ADCs, RICs, TCEs, and/or CAR‐Ts, the integrative nature of computational pathology approaches provides opportunities for the implementation of combinatorial biomarkers to guide patient selection and treatment response prediction. For instance, from the H&E and target IHC WSIs, it is possible to concurrently derive the quantification of the target on the cell membrane of cancer cells, as well as a quantification and spatial distribution analysis of immune cells. These multimodal combined approaches herald a new era of biomarkers that more holistically assess the phenotypic characteristics of the cancer cells and the immune TME.

## 5 The future of precision medicine with digital pathology

With the pace of development of ADCs, and the excitement surrounding the development of RIC, TCEs, and CAR‐Ts for solid malignancies, it is plausible that in the not‐so‐distant future every cancer patient will require the quantification of at least one target, if not multiple targets, to define their optimal treatment path. Computational pathology offers the opportunity to democratize the access to expert diagnostic pathology expertise and to predictive biomarkers, overcoming the challenges posed by the limited availability of diagnostic pathologists, particularly in underserved regions. Despite the promise of computational pathology and the exciting solutions already developed, the vast majority of diagnostic pathology departments in the world have not undergone the digital pathology transformation required for the deployment of these transformational approaches. In fact, only a small minority of academic department and < 5% of the community hospitals in the USA have fully digital diagnostic workflows. The digital transformation will inevitably require the engagement of stakeholders across disciplines and industries, as well as the education of the next generation of pathologists fully conversant in the deployment of computational pathology methods in clinical practice.

We would also contend that the opportunities offered by generative AI and by pathology‐focused as well as multimodal foundation models will transform, once again, the landscape of biomarkers that can be derived from H&E and IHC WSIs. In fact, recent studies demonstrate that foundation models, self‐supervised AI models trained on pre‐trained on large, diverse datasets of pathology images, can perform as well as trained diagnostic pathologists in the execution of multiple common tasks performed by pathologists, and that these models can infer transcriptomic profiles and the presence of specific genomic alterations from H&E sections with no‐to‐minimal additional training [9, 10]. The potentials of these novel AI approaches are immense and one could posit that such approaches may enable the development of comprehensive assessment of targets and the TME from a single H&E and a limited number of IHC preparations.

## 6 Conclusion

Advancements in digital and computational pathology are setting new standards in the precision, accuracy, and reproducibility of cancer biomarkers measurement. These approaches can potentially provide an unprecedented level of information about therapeutic targets and TME, biological features that may prove essential for the effective deployment of ADCs, RICs, TCEs, and CAR‐Ts. With the unprecedented developments in generative AI and foundation models, we anticipate an even greater number of fit‐for‐purpose diagnostic solutions, biomarkers for patient selection and treatment response prediction, as well as end‐to‐end predictive solutions to be developed. Solutions to enable the digital pathology and, subsequently, computational pathology revolution are urgently needed, and will require a close partnership involving pathologists, oncologists, diagnostic companies, the pharmaceutical industry, payers, and regulatory agencies [11]. As we move forward, however, the integration of these synergistic and transformational technologies into routine clinical practice will have a profound impact on oncology and pathology, heralding a new era of personalized medicine.

## Conflict of interest

JSR‐F, MS, and SG are employees of AstraZeneca. AK and HS are employees of AstraZeneca Computational Pathology GmbH.

## Author contributions

JSR‐F, MS, AK, and HS performed the literature review; JSR‐F and MS developed the first draft of the manuscript, which was reviewed, edited, and approved by all authors.
